# Therapeutic Potential of *Solenopsis invicta* Venom: A Scoping Review of Its Bioactive Molecules, Biological Aspects, and Health Applications

**DOI:** 10.3390/biom14121499

**Published:** 2024-11-24

**Authors:** Mario Dioguardi, Stefania Cantore, Diego Sovereto, Lorenzo Sanesi, Angelo Martella, Lynn Almasri, Gennaro Musella, Lorenzo Lo Muzio, Andrea Ballini

**Affiliations:** 1 Department of Clinical and Experimental Medicine, University of Foggia, Via Rovelli 50, 71122 Foggia, Italy; diego_sovereto.546709@unifg.it (D.S.); lorenzo.sanesi@unifg.it (L.S.); gennaro.musella@unifg.it (G.M.); lorenzo.lomuzio@unifg.it (L.L.M.); andrea.ballini@unifg.it (A.B.); 2 Department of Precision Medicine, University of Campania Luigi Vanvitelli, Via De Crecchio, 7, 80138 Naples, Italy; 3 DataLab, Department of Engineering for Innovation, University of Salento, 73100 Lecce, Italy; angelo.martella@unisalento.it; 4 King’s College London, University of London, Strand, London WC2R 2LS, UK; lynn.almasri@outlook.com

**Keywords:** *Solenopsis invicta*, RIFA, tumour, antimicrobial, cancer, angiogenesis, venom, head and neck squamous cell carcinomas (HNSCCs), drug discovery, candida

## Abstract

*Solenopsis invicta,* a South American ant species from the Formicidae family (subfamily Myrmicinae), has recently established a stable settlement in Europe, raising public health concerns due to its venomous stings. The venom of *S. invicta* is rich in bioactive molecules, particularly piperidine alkaloids such as solenopsin A and peptides (Sol 1–4). These compounds have been implicated in various health applications, including antimicrobial, anti-inflammatory, and antitumour activities. While previous reviews have focused on the ecological and allergenic risks posed by *S. invicta,* this scoping review aims to evaluate the potential therapeutic uses of *S. invicta* venom by summarizing existing scientific evidence and providing a novel synthesis of recent research on its bioactive components. Furthermore, this study, by describing the unique biological aspects of *S. invicta*, provides an overview of its direct impact on public health, highlighting new findings on the venom’s role in inhibiting bacterial biofilm formation and modulating cancer growth pathways through gene regulation. A search of databases (PubMed, Scopus, Science Direct, and Cochrane Library) identified 12,340 articles, from which 11 studies met the eligibility criteria. These studies included seven microbiological investigations and four studies on tumour cell lines and animal models. The findings suggest that *S. invicta* venom could inhibit biofilm formation, combat fungal infections, and suppress tumour growth. However, further research, including clinical trials, is required to fully elucidate the safety and efficacy of these bioactive molecules in human medicine, for their potential use in drug discovery to counteract several diseases, including cancer.

## 1. Background

Many arthropods, including numerous insect species, produce venoms with a wide range of pharmacological and biochemical properties. These venoms can have both harmful and potentially beneficial effects on human health, depending on the insect species and venom composition [1].

In terms of public health, insect stings and bites can pose a threat to the population due to the presence of venom, which can cause local or systemic allergic reactions, anaphylaxis, and even death in severe cases [2]. Additionally, some arthropod species can transmit infectious diseases through bites or stings, increasing the risk of pathogens spreading among humans [3].

However, it is also important to consider the potential of insect venoms for beneficial biomedical applications. For example, certain substances found in insect venoms have demonstrated antimicrobial [4], anti-inflammatory [5], analgesic [6], and antitumour properties [7]. These properties can be exploited to develop new drugs for treating a range of medical conditions, including bacterial and viral infections, chronic inflammation, neuropathic pain [8], and cancers [9,10], such as head and neck squamous cell carcinomas (HNSCCs).

Considering only recent studies (2023–2024), the field of insect venom application in medicine is highly active, and numerous studies on hymenopteran venom have reported various therapeutic effects [11].

Anti-inflammatory and antiviral activity against the herpes simplex viruses HSV-1 and HSV-2 has been observed for peptides from melittin of *Apis mellifera* and *Apis florea* [12]. Additionally, peptides from the venom of *Vespa magnifica* have been reported to have effects on rheumatoid arthritis [13].

Among insects, the venom from certain ants has also been investigated. A recent study on the venom of *Dinoponera quadriceps*, specifically focusing on three dinoponeratoxins, suggested potential anticonvulsant action in epilepsy treatment [14]. Furthermore, the peptide bicarinalin, isolated from the venom of *Tetramorium bicarinatum* ants, has been studied as an active substance against *Helicobacter pylori*, which causes several gastric diseases [4].

*Paraponera clavata* is reported to have peptides with analgesic effects [6], and venom extracted from *Polyrhachis lamellidens*, in addition to having anti-inflammatory effects [5], has been studied as a potential agent for treating Alzheimer’s disease [15].

The venom of *Solenopsis invicta*, an insect, has been investigated for its antitumour and antimicrobial effects [16].

### 1.1. Biological Aspects of Solenopsis invicta

**Figure 1:**
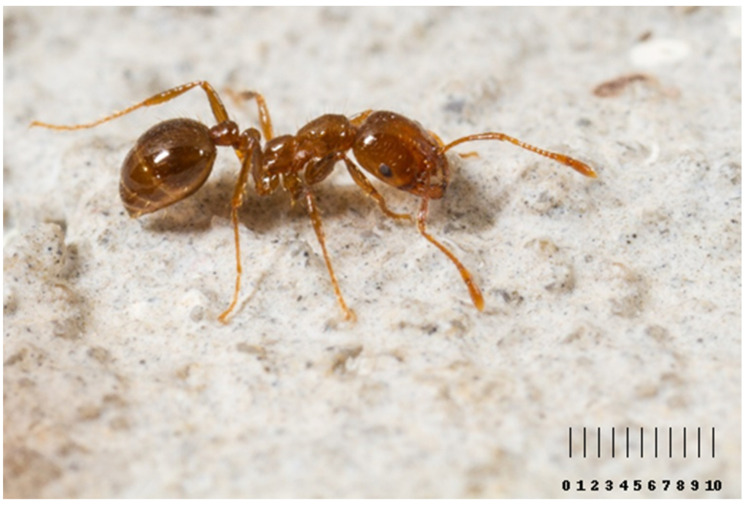
*Solenopsis invicta* worker. The workers exhibit pronounced polymorphism [20], with lengths ranging from 1.5 mm to 4 mm, while the queens measure between 6 mm and 8 mm in length. The ant is identifiable by its reddish-brown colour with darker tones on the gaster and the presence of a stinger at the end of its gaster. *Solenopsis invicta* accumulates venom in its poison sac and convoluted gland, located within the abdomen, primarily near the stinger [21] (https://www.shutterstock.com/it/image-photo/red-imported-fire-ant-solenopsis-invicta-181051103 (access on 21 June 2024)) (in millimetre scale, 10 ≅ 1 mm).

*Solenopsis invicta* belongs to the Formicidae family (the *Myrmicinae* subfamily). They are reddish-brown in colour and are present in different sizes, categorized as small, medium, and large, with lengths ranging from 2 mm to 4 mm [17] (Figure 1).

*Solenopsis invicta* lives in colonies that can accommodate 80,000 to 400,000 individuals per colony and can be either monogynous (with a single queen) or polygynous (with multiple queens). A mature colony develops in 6–8 months, followed by the development of winged reproductive adults. The nuptial flight of these winged adults occurs in spring and summer, with a yearly total of 4000 to 6000 winged adults [18]. The nests, which can range from 60 to 250 per hectare, are identifiable as mounds of soil, standing at 120 cm in height, and lack a direct entrance hole (the ants enter and exit through lateral tunnels that can extend up to 30 m) [18,19].

The presence of a stable settlement comprising approximately 88 nests of *Solenopsis invicta,* a species classified as one of the worst invasive alien species, has been reported and documented for the first time near Syracuse, Sicily, Italy [22]. This represents the first stable settlement of this insect in Europe. Previously, a nest was identified in the soil of Ficus plants imported from the United States to the Netherlands [23]. Furthermore, live worker specimens have been intercepted on multiple occasions in containers containing imported fruits and fresh products in Europe [23].

Its origin is South America, and it was first identified in 1917 as *Solenopsis saevissima wagneri*. It was subsequently recognized as a distinct species in 1972 by Buren and named *Solenopsis invicta* [22]. It began to spread in the 1920s in Alabama, USA, likely because of the darker and less invasive *Solenopsis richetri* [24]. In the 1950s, it proliferated widely in a lighter (red) form, identified by Buren as *Solenopsis invicta*, commonly known as the red imported fire ant (RIFA), confirming the dual introduction of Solenopsis species ants in the USA [25]. In 2001, it also arrived in Brisbane, Australia [26], and in 2003, it arrived in Taiwan. Between 2001 and 2006, three *Solenopsis invicta* infestations occurred in New Zealand, which is currently the only country that has successfully eradicated *Solenopsis invicta* [27,28].

In general, *Solenopsis invicta* are carefully considered due to their aggressive behaviour (biting) and venomous stings. This venom is composed of water-insoluble alkaloids and soluble peptides, which are responsible for anaphylactic reactions [29].

Venom plays a role in prey capture, defence, communication [30], and nutrition [31], and it can also serve as an attractant for female phorid flies. These flies utilize *Solenopsis invicta* as a living incubator for their larvae, which feed on the ant’s body [32].

**Figure 2:**
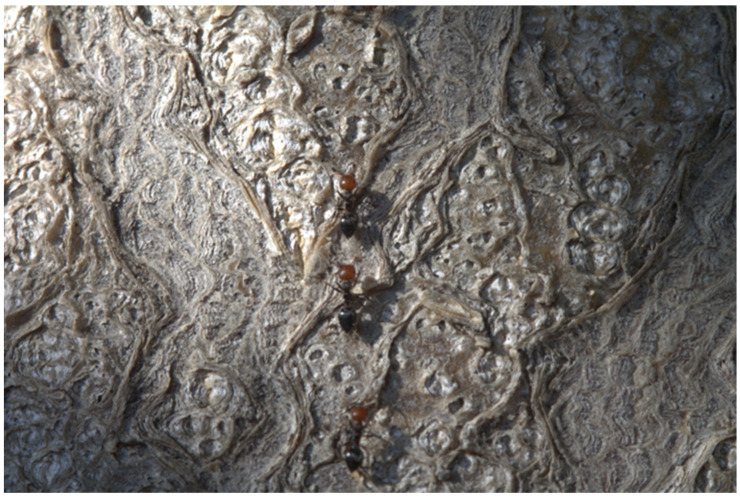
Native Mediterranean ant species: Workers in a row of *Crematogaster scutellaris* on the trunk of *Ailanthus altissima*; photo taken by Mario Dioguardi and Diego Sovereto at the soil surrounding University of Foggia Dental Clinic, Italy. The impact on the ecosystem results in reduced native biodiversity with the potential to displace native ant species [34,35] and attacks other invertebrates [36], reducing their populations. This invasive species can also alter the physical and chemical properties of soils through structural modifications and nutrient accumulation during nest construction [37]. Moreover, they can cause damage to human infrastructure in urban areas, affecting sidewalks, cables, and wires.

The primary impact on the economic production system is primarily through direct damage to plants, causing agricultural losses, especially to tubers, roots, flowers, fruits, and seeds (damage to *S. tuberosum, Brassica oleracea* var. capitata, and *Solanum melongena*). It also harms the regrowth of fruit trees (such as citrus) and is influenced by mutualistic relationships with other pests (Figure 2), such as aphids and scale insects [33].

The impact on farm animals mainly concerns attacks on newborns or sick or weak animals that cause death or blindness (they can sting near the eyes); these animals can also infest foodstuffs by biting and stinging animals that go to eat, which then stops feeding [38].

### 1.2. Impact Human Health

Furthermore, stings can directly impact human health by causing anaphylactic crises, occasional convulsions [39], and potentially death [40], considering that in some areas of the USA and China, at least 30% of the population are stung annually [41].

One of the pivotal aspects that renders it a threat to public health lies in its aggressiveness towards other species, which poses a significant issue for ecological balance and human health [42]. Specifically, it represents a greater risk than stings from other hymenopterans, owing to its venomous and painful stings [43]. Its size, much smaller than wasps and bees, coupled with its propensity to attack large groups (with colonies of sizable proportions reaching up to 200,000 individuals), heightens the risk of severe harm. Moreover, a single worker of *Solenopsis invicta* can sting repeatedly without breaking its stinger, potentially causing more serious damage than that inflicted by an *Apis mellifera* sting [44]. Such stings may induce pustules, localized reactions, and, in some instances, anaphylaxis and even death [45].

The lesions may endure for an extended period and are not always confined to the skin. Retrospective case analyses of corneal damage from *Solenopsis invicta* stings have shown that such stings can result in long-lasting corneal lesions that are unresponsive to conventional topical treatment with antibiotics and steroids [46].

**Figure 3:**
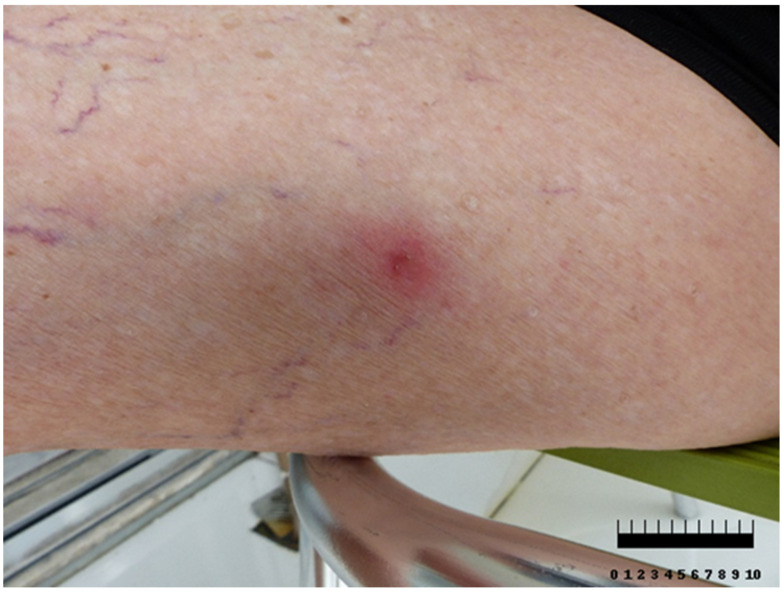
Red imported fire ant (*Solenopsis invicta):* the bite and puncture lesion are highlighted. The area appears erythematous with the presence of edema (https://www.shutterstock.com/it/image-photo/red-imported-fire-ant-bite-solenopsis-1039911532 (access on 21 June 2024)). (In cementers scale, 1 ≅ 1 cm).

*Solenopsis invicta* has four teeth that it uses to bite while arching its back, and it stings with a posterior stinger, rotating its head in a circular pattern, resulting in multiple stings. At the inoculation site, pustules form due to the action of the alkaloids present in the venom (the venom differs from that of other hymenoptera, which consist of hydrosoluble peptide solutions). This is followed by a cellular infiltration of eosinophils and neutrophils with fibrin deposition and basal necrosis within 24 h, and it is important to minimize secondary bacterial infection. In humans, the inoculation site produces a burning pain as a symptom and can cause a broad range of inflammatory reactions (Figure 3). In such cases, the lesions appear erythematous, edematous, and indurated with intense itching that can last up to 72 h [47].

### 1.3. Venom and Bioactive Molecules

**Figure 4:**
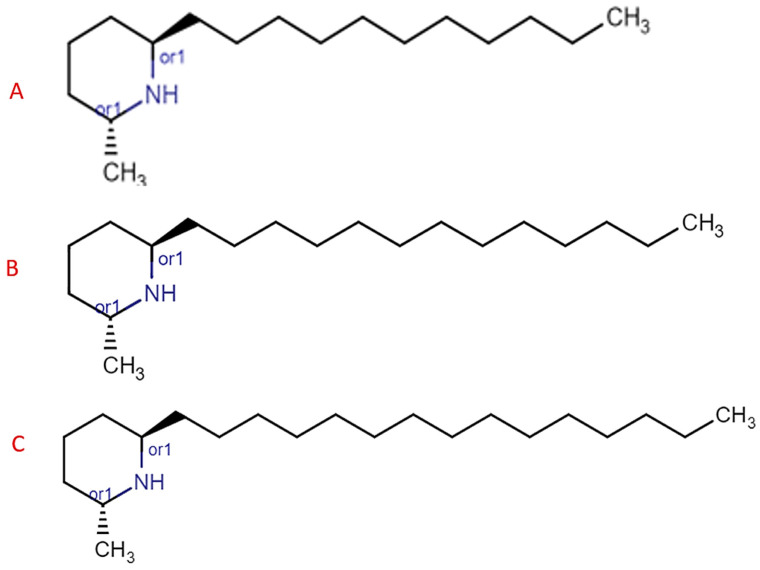
(**A**): Trans-2-Meth-6-undecyl piperidine (solenopsin A), (**B**): trans-2-methyl-6-tridecylpiperidine (solenopsin B), (**C**): trans-2-methyl-6-pentadecylpiperidine (solenopsin C); the figures of the molecules were obtained via the use of the online software https://chemicalize.com/app/calculation on 16 June 2024 starting from the IUPAC formula.

The venom of *Solenopsis invicta* is rich in alkaloids (2-methyl-6-alkyl or alkenyl piperidines) [48] and some peptides, which are responsible for symptoms and anaphylaxis but can be biologically active with many potential pharmacological activities [49]. Specifically, the venom is composed of 95% insoluble piperidine alkaloids, commonly referred to as solenopsins. These compounds are classified based on the length of the R side chain (alkyl) present at position 6 on the piperidine ring (A: C11, B: C13, C: C15, and D: C17), as reported in Figure 4 (further chemical formulas of the alkaloids present in the venom of *Solenopsis invicta* have been reported in Supplementary Materials S1). The remaining 5% consisted of water-soluble components, with 0.1% being highly allergenic proteins. A single sting from *Solenopsis invicta* contains only 10–100 ng of protein, which can trigger anaphylactic reactions comparable to those caused by wasps and bees. These proteins are named Sol (1–4), with Sol 2 and Sol 3 being abundant and Sol 2 and Sol 4 being exclusive to fire ants [50].

Some studies have described its potential positive effects in the symptomatic treatment of psoriasis [51] and malaria [49,52]. A recent study conducted by Mo et al. [16] identified a specific alkaloid, solenopsin-A, in the venom of *Solenopsis invicta*, which demonstrated antiangiogenic activity against neoplasms, suggesting its potential use as an antitumour agent, similar to other invertebrate venoms [10].

The purpose of this scoping review is to compile and summarize scientific studies on the therapeutic potential of *Solenopsis invicta* venom. This review aims to evaluate whether the alkaloids and peptides found in the venom offer meaningful benefits in the treatment and prevention of human diseases.

## 2. Methods

### 2.1. Protocol and Registration

The scoping review was conducted following the PRISMA-ScR checklist (PRISMA Extension for Scoping Reviews), as outlined by Tricco et al. [53]. The scoping review was registered on INPLASY (International Platform of Registered Systematic Review and Meta-analysis Protocols) under the registration number INPLASY202490103, at http://doi.org/10.37766/inplasy2024.9.0103. Further information on the recording protocol is provided in Supplementary Materials S2.

### 2.2. Eligibility Criteria

All studies related to *Solenopsis invicta* venom in the context of human disease treatment and prevention were considered potentially eligible. No restrictions were applied based on the publication year or language, provided that an English-language abstract was available. The inclusion criteria encompassed all clinical and preclinical studies (in vitro, in vivo) investigating the therapeutic effects and potential medical applications (antibacterial, antitumour, and anti-inflammatory activities). The exclusion criteria for studies were as follows: studies with weak or unclear methodological design, studies that were not peer-reviewed or published in sources of dubious reliability, and duplicate studies or those with redundant information already included in other studies. Literature reviews were excluded and used solely as sources for bibliographic research and informative purposes.

### 2.3. Information Sources

The search was conducted across three databases (PubMed, Scopus, and Science Direct) and a registry (Cochrane Library). Additionally, a grey literature search was performed on Google Scholar and OpenGray (DANS EASY Archive). Furthermore, it should be noted that textbooks were identified and searched alongside other records (reports, articles, studies, doctoral theses, and proceedings) through databases such as ScienceDirect and Google Scholar, which index them (partially also PubMed and Scopus). Potentially relevant articles were also sought within the references of literature reviews on *Solenopsis invicta.*

The search was carried out from 1 September 2023 to 10 October 2023, with a final update of the records identified on 1 July 2024.

On 3 March 2024, an additional bibliographic search was conducted accessing other databases: EBSCO, Web of Science, and LILACS.

### 2.4. Search

The authors responsible for the research of studies (M.D.) used the following keywords in the databases: Solenopsis Invicta OR RIFA OR Hymenoptera Formicidae OR fire ant OR solenopsin. The keywords used on PubMed are listed below.

✓ Search: fire ant OR solenopsis OR RIFA Sort by: Most Recent
  “fire ants” [MeSH Terms] OR (“fire” [All Fields] AND “ants” [All Fields]) OR “fire ants” [All Fields] OR (“fire” [All Fields] AND “ant” [All Fields]) OR “fire ant” [All Fields] OR “solenopsis” [All Fields] OR “RIFA” [All Fields]
  Translationsfire ant: “fire ants” [MeSH Terms] OR (“fire” [All Fields] AND “ants” [All Fields]) OR “fire ants” [All Fields] OR (“fire” [All Fields] AND “ant” [All Fields]) OR “fire ant” [All Fields]
✓ Search: Hymenoptera Formicidae Sort by: Most Recent
  (“hymenoptera” [MeSH Terms] OR “hymenoptera” [All Fields]) AND (“ants” [MeSH Terms] OR “ants” [All Fields] OR “formicidae” [All Fields])
  Translations
  Hymenoptera: “Hymenoptera” [MeSH Terms] OR “Hymenoptera” [All Fields]
  Formicidae: “ants” [MeSH Terms] OR “ants” [All Fields] OR “formicidae” [All Fields]
✓ Search: solenopsin Sort by: Most Recent
  “solenopsin” [All Fields]

### 2.5. Search Strategy of Sources of Evidence

The search for potentially eligible studies was carried out by two reviewers (M.D. and S.C.), with a third reviewer (A.B.) tasked with making inclusion decisions in case of conflicts.

After jointly deciding on eligibility criteria, the databases to use, and the keywords, the two reviewers independently conducted the search, reporting the number of records retrieved for each keyword and each database used. Duplicate records from different databases were removed using EndNote software (ver. 9.3), and overlapping studies that could not be loaded into EndNote were manually removed after the screening phase. The screening and inclusion of studies from the obtained records were also independently conducted, with a subsequent comparison of the included studies between the two reviewers.

### 2.6. Data Charting Process, Data Items, Synthesis of Results

The data to be extracted were predetermined by the authors and included the primary author, publication date, study location, study type (microbiological, in vivo, or cell line type), specific disease under examination, active ingredient of *Solenopsis invicta* venom used, and key findings. Data extracted from the studies were independently recorded in Word tables by the two reviewers and later compared. The obtained data are presented in tables and included in the Results section of this manuscript.

## 3. Results

### 3.1. Selection of Sources of Evidence

The searches across Science Direct, SCOPUS, PubMed, and the Cochrane Library yielded a total of 13,336 bibliographic sources. After removing duplicates, 12,340 unique sources remained. Among them, 79 articles were potentially eligible, with only 11 fully meeting the eligibility criteria.

Additionally, analyses of grey literature (http://www.opengrey.eu, accessed on 9 October 2023, DANS EASY Archive, and Google Scholar) and previous systematic reviews did not identify any additional studies to include in the qualitative assessment (Figure 4).

On 3 March 2024, using “*Solenopsis invicta*” as the keyword, an additional bibliographic search was conducted accessing other databases: EBSCO, Web of Science, and LILACS.

**Figure 5:**
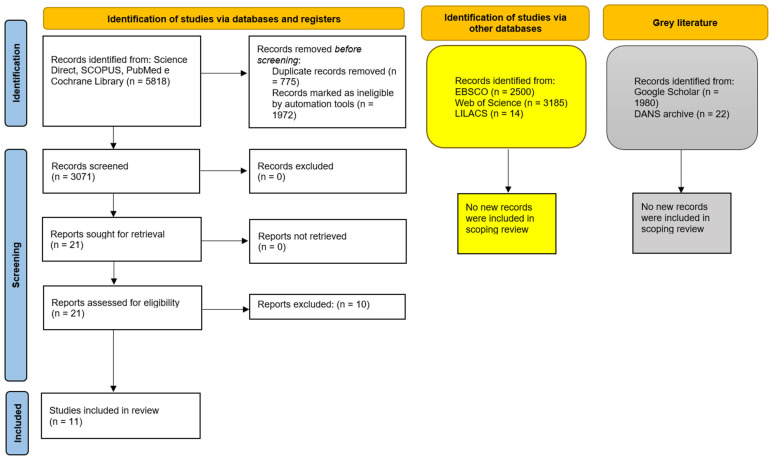
The entire selection and screening procedures are described in the PRISMA flowchart. The search was carried out from 1 September 2023 to 10 October 2023, with a final update of the records identified on 1 July 2024; in the yellow boxes, the number of records identified on EBSCO, Web Of Science, and LILACS as of 3 March 2024 are reported; in the grey boxes, the records identified on Google Scholar (using the keyword: *Solenopsis invicta* AND cancer) and on OPENGREY. DANS EASY Archive using the keyword “solenopsis” are reported.

The entire procedure for the identification, selection, and inclusion of studies is outlined in the flowchart depicted in Figure 5.

### 3.2. Characteristics of Sources of Evidence and Results of Individual Sources of Evidence

A total of 11 studies were included in the scoping review:

✓ Seven microbiological in vitro studies: Blum et al. (1958) [54], Jouvenaz et al. (1972) [55], Sullivan et al. (2009) [56], Park et al. (2008) [57], Carvalho et al. (2019) [58], Yan et al. (2017) [59], and Silva et al. (2020) [60];
✓ Three in vitro studies on tumour cell lines and one animal model study: Arbiser et al. (2007) [61], Uko et al. (2019) [62], Karlsoon et al. (2015) [63], and Arbiser et al. (2017) [64].

The microbiological studies included different microorganisms, including bacteria (e.g., *Escherichia coli*, *Enterococcus faecalis*, *Pseudomonas aeruginosa*), fungi (*Candida albicans*, *Aspergillus fumigatus*), and protozoa (*Trypanosoma brucei rhodesiense*, *Trypanosoma cruzi*, and *Leishmania donovani*). Table 1 provides detailed data on all the extracted information and the microbial species tested in vitro with *Solenopsis invicta* venom.

The primary diseases addressed in the studies, upon which cell lines and animal models were tested, were primarily related to oncologic conditions (melanoma, squamous carcinoma, renal carcinoma, and lung cancer) and hyperproliferative skin disorders such as psoriasis. Table 2 contains comprehensive data extracted from studies on tumour cell lines and/or in vivo models.

Table 2 reports studies that have also investigated toxicity on human cell lines including tumour lines, including the following: human lung tumour cells [62], human A375 melanoma cells [63], human A2058 melanoma cells [63], primary human melanocytes [63], primary human keratinocyts, HaCaTs (immortalized human keratinocytes) [63], and human UM-SCC1A squamous carcinoma cells [63].

We further assessed the selectivity of *Solenopsis invicta* venom alkaloids and peptides, focusing on their potential cytotoxicity towards healthy human cells. While several studies indicate promising therapeutic actions, the selectivity of these compounds remains an area for further investigation. In silico tools, such as those discussed in recent studies [65,66,67] offer new approaches for predicting the toxicity and selectivity of venom-derived compounds, enabling researchers to assess safety profiles efficiently.

These methods could be particularly useful for advancing the therapeutic application of *Solenopsis invicta* venom compounds by providing initial toxicity data and guiding laboratory testing.

In fact, in silico studies have investigated the allergenic cross-reactivity of fire ant SOL peptides providing reliable insights to design future therapeutic strategies [68].

The excessive heterogeneity of the data observed here reflects differences in the approaches taken by various studies. Firstly, the alkaloid compounds (including the various forms of solenopsin and its analogues, which numbered over 17, as shown in Table 3) and peptides investigated varied significantly across studies (Table 3 and Table 4), as did the experimental methods employed.

The microorganisms analyzed also exhibited considerable diversity, except for a few that were studied in multiple investigations, such as *Pseudomonas aeruginosa* [55,56,57], *Staphylococcus aureus* [55,56,59], *Escherichia coli* [54,56], and *Streptococcus pyogenes* [54,55]. Despite this, the MIC range (Table 2) showed such high variability that aggregating data into a meta-analysis without incurring bias was not feasible.

For instance, MIC values varied widely, from 20.0 ± 0 μg/mL for *Candida albicans* up to 0.5 mg/L for *Pseudomonas aeruginosa* [56], and for *Pseudomonas fluorescens*, MIC values ranged from 370.4 μg/mL to as high as 5000 μg/mL [58].

**Table 1:** In vitro microbiological studies of compounds 1, 2, and 3 (2-methyl-6-pentadecyl-Δ1,6-piperideine (1), 2-methyl-6-tetradecyl-Δ1,6-piperideine (2), and 2-methyl-6-hexadecyl-Δ1,6-piperideine (3)).

First Author, Date	Country	Ant	Microorganisms	Tested Product and Concentration	Method	Results
**Blum et al., 1958** [54]	USA	*Solenopsis* *saevissima* var. *richteri*	*Micrococcus pyogenes*, *Streptococcus pyogenes*, *Escherichia coli*, *Lactobacillus casei*	1/50 dilution of the venom extract;	Paper disc-diffusion	antibiotic activity
**Jouvenaz et al., 1972** [55]	USA	*Solenopsis invicta*	*Streptococcus salivariu, Streptococcus pyogenes*, *Streptococcus equisimilis*, *Streptococcus Faecalis*, *Staphylococcus epidermidis*, *Staphylococcus aureus*, *Bacillus pulvifacienis*, *Bacillus thuringientsis*, *Shigella flexneri*, *Shigella boydii*, *Shigella sonnei*, *Salmonella Typhimurium*, *Salmonella paratyphi*, *Salmonella schottmuelleri*, *Salmonella enteritidis*, *Escherichia coli*, *Proteus* spp., *Klebsiella pneumonziae*, *Alcaligenes faecalis* and *Pseudomonas aeruiginosa*	1:1000 aqueous solution of solenopsin HCl, applied to 6.0 mm paper discs and air-dried at 37 °C.	Paper disc-diffusion	Inhibition: *Staphylococcus aureus* and *Escherichia coli*
**Sullivan et al., 2009** [56]	USA	*Solenopsis invicta*	*Streptococcus pneumoniae, Staphylococcus aureus*, *Enterococcus faecalis*, *Escherichia coli*, *Stenotrophomonas maltophilia* and *Pseudomonas aeruginosa*	Lyophilized venom alkaloids (1 mg/L) were diluted in a 5% solution of (2-hydroxypropyl)-β-cyclodextrin. Increasing concentrations of alkaloid were prepared by dilution of the stock 1 mg/L cyclodextrin (5%) in Mueller-Hinton broth.	MIC (Minimum Inhibitory Concentration) using broth dilution method	Inhibition: *Streptococcus pneumoniae*, *Staphylococcus aureus*, *Enterococcus faecalis* and *Stenotrophomonas maltophilia*
**Park et al., 2008** [57]	USA	*Solenopsis invicta*	*Pseudomonas aeruginosa*	Cells were diluted into fresh Luria–Bertani containing solenopsin (50 µmol/L)	Quorum-Sensing (QS) Signalling	Solenopsin A, suppressed QS signalling in *Pseudomonas aeruginosa*
**Carvalho et al., 2019** [58]	Brazil	*Solenopsis invicta*	*Pseudomonas fluorescens*	Solenopsins at concentrations of 500, 750, 1000, and 5000 μg/mL applied to 6 mm sterile filter paper discs	MIC (Minimum Inhibitory Concentration)	Inhibition: *Pseudomonas fluorescens*
**Yan et al., 2017** [59]	China	*Solenopsis invicta* and *Solenopsis richteri*	*Cryptococcus neoformans, Candida albicans, Leishmania donovani promastigotes, Trypanosoma brucei, Aspergillus fumigatus,* antibacterial activity against methicillin-resistant *Staphylococcus aureus* (MRSA) and vancomycin-resistant *Enterococcus faecium*	Compound 1a: IC50 of 6.6 μg/mL (*Cryptococcus neoformans*) and 12.4 μg/mL (*Candida albicans*); IC50 value of 19.4 μg/mL (*Enterococcus faecium)* Compound 1–3: IC50 of 5.0−6.7 μg/mL (*Leishmania donovani promastigotes*) and 2.7−4.0 μg/mL *Trypanosoma brucei*)	MIC (Minimum Inhibitory Concentration)	Antifungal activity against *Cryptococcus neoformans* and *Candida albicans*, antiprotozoal activity against *Leishmania donovani promastigote*s and *Trypanosoma brucei*
**Silva et al., 2020** [60]	Brazil	*Solenopsisinvicta* and *Solenopsis saevissima*	*Trypanosoma brucei rhodesiense* and *Trypanosoma cruzi*	Solenopsins at concentrations ranging from 0.1 to 384.0 µM tested over 16 days	MIC (Minimum Inhibitory Concentration)	Inhibition: *Trypanosoma brucei rhodesiense* and *Trypanosoma cruzi*

**Table 2:** The main data extracted from the included studies are reported regarding the effects of alkaloids derived from the venom of *Solenopsis invicta* on cell lines and experimental animal models.

First Author, Date	Country	Active Compound	Cell Lines, Animal Model	Main Results
**Arbiser et al., 2007** [61]	USA	Solenopsina-A	786-O cells, in vivo embryonic zebrafish	Inhibition of angiogenesis
**Uko et al., 2019** [62]	USA	Solenopsin Analogue	Human lung tumour cells, WB-ras rat liver epithelial	Inhibition of angiogenesis
**Karlsoon et al., 2015** [63]	USA	Solenopsin-A	Human A375 melanoma cells, human A2058 melanoma cells, immortalized murine endothelial SVR cells, primary human melanocytes, primary human keratinocyts, HaCaTs (immortalized human keratinocytes), murine embryonic NIH3T3 fibroblasts, and human UM-SCC1A squamous carcinoma cells	Biological activity similar to ceramide in human melanoma cells
**Arbiser et al., 2017** [64]	USA	Solenopsin Analogue	Mouse KC-Tie2	Significant decreases in acanthosis and hyperkeratosis

**Table 3:** Main solenopsin compounds and derivatives investigated in the included studies. The names of the compounds and the analogues of solenopsin are reported in the included studies; however, they do not always reflect the IUPAC nomenclature. Additionally, synonyms are present within the table. For clarity, we have retained the original names as reported in the studies (the cis or trans form of the compound is not always specified). The analogues of solenopsin are listed from S1 to S17, which generally differ according to the R group. It should be noted that the analogues, even if they bear the same name, do not always correspond to the same compound, as they may vary from study to study.

First Author, Date	Compound
**Blum et al., 1958** [54]	Venom Ant
**Jouvenaz et al., 1972** [55]	trans-2- methyl-6-n-tridecylpiperidine (solenopsin B)
	trans-2-methyl-6-(cis-4-tridecenyl)-piperidine (dehydrosolenopsin B)
	trans-2-methyl-6-n-pentadecylpiperidine (solenopsin C)
	trans-2-methyl-6-(cis-6-pentadecyl)-piperidine (dehydrosolenopsin C)
**Arbiser et al., 2007** [61]	Solenopsin A, (analogue: S2–17)
**Sullivan et al., 2009** [56]	(+)-solenopsin (Sol) A
	(2R, 6R)-solenopsin A
	(2S, 6S)-solenopsin B
	(+)-isosolenopsin A
	(2S, 6R)-isosolenopsin A
	(2R, 6S)-isosolenopsin A
	(+)-isosolenopsin B
	(2S, 6R)-isosolenopsin B
	(2R, 6S)-isosolenopsin B
**Park et al., 2008** [57]	Solenopsin A, (analogue: S1–5)
**Karlsoon et al., 2015** [63]	(+)-Solenopisin A
	(-)-Solenopisin A
	Solenopsin analogue: S11: 2,4 dimethyl-6-nonadecylpiperinide
	Solenopsin analogue: S12–15
**Arbiser et al., 2017** [64]	Solenopsin analogue: S12
	Solenopsin analogue: S14
**Yan et al., 2017** [59]	2-methyl-6-pentadecyl-Δ1,6-piperideine
	2-methyl-6-tetradecyl-Δ1,6-piperideine
	2-methyl-6-hexadecyl-Δ1,6-piperideine
**Uko et al., 2019** [62]	Solenopisin A
	Solenopsin analogue compounds B: 2-Dodecylsulfanyl-1, -4, -5, -6-tetrahydropyrimidine
	Solenopsin analogue compounds c: [(dodecylsulfanyl)(methylamino)methyl](methyl)amine
	Solenopsin analogue compounds d: 2-(dec-9-en-1-yl)-3-ethyl-1,3-oxazolidine
**Carvalho et al., 2019** [58]	cis-2-Me-6-Tridecyl-Piperidine
	trans-2-Me-6-Tridecenyl-Piperidine
	trans-2-Me-6-Tridecyl-Piperidine
	cis-2-Me-6-Pentadecyl-Piperidine
	trans-2-Me-6-Pentadecenyl-Piperidine
	trans-2-Me-6-Pentadecyl-Piperidine
**Silva et al., 2020** [60]	Isosolenopsin A: cis-2-Me-6-undecyl piperidine
	Solenopsin A: trans-2-Me-6-undecyl piperidine
	Dehydrosolenopsin B: trans-2-Me-6-tridecenyl piperidine
	Solenopsin B: trans-2-Me-6-tridecyl piperidine
	Dehydrosolenopsin C: trans-2-Me-6-pentadecenyl piperidine
	Solenopsin C: trans-2-Me-6-pentadecyl piperidine

**Table 4:** Table outlining the chemistry and properties of alkaloids, specifically solenopsins, as reported in the included studies. The calculations for exact mass, molar mass, chemical formula, and chemical composition were performed using the online software at https://chemicalize.com/app/calculation on 16 June 2024, based on the IUPAC name or molecular structure depicted in the included studies, if not provided in the original publications.

	Molar Mass	Exact Mass	Formula	Composition	Reference
**cis-2-Meth-6-undecyl piperidine**	253.474 g/mol	253.276950131 Da	C_17_H_35_N	C (80.56%), H (13.92%), N (5.53%)	Silva et al., 2020 [60]
**trans-2-Meth-6-undecyl piperidine**	253.474 g/mol	253.276950131 Da	C_17_H_35_N	C (80.56%), H (13.92%), N (5.53%)	Silva et al., 2020 [60], Uko et al., 2019 [62], Arbiser et al., 2007 [61]
**trans-2-Meth-6-tridecenylpiperidine**	279.512 g/mol	279.292600195 Da	C_19_H_37_N	C (81.65%), H (13.34%), N (5.01%)	Silva et al., 2020 [60], Carvalho et al., 2019 [58], Jouvenaz et al., 1972 [55]
**trans-2-Meth-6-tridecyl piperidine**	281.528 g/mol	281.30825026 Da	C_19_H_39_N	C (81.06%), H (13.96%), N (4.98%)	Silva et al., 2020 [60], Carvalho et al., 2019 [58], Jouvenaz et al., 1972 [55]
**trans-2-Meth-6-pentadecenylpiperidine**	307.566 g/mol	307.323900324 Da	C_21_H_41_N	C (82.01%), H (13.44%), N (4.55%)	Silva et al., 2020 [60], Carvalho et al., 2019 [58], Jouvenaz et al., 1972 [55]
**trans-2-Meth-6-pentadecylpiperidine**	309.582 g/mol	309.339550389 Da	C_21_H_43_N	C (81.47%), H (14.00%), N (4.52%)	Silva et al., 2020 [60], Carvalho et al., 2019 [58], Jouvenaz et al., 1972 [55]
**cis-2-Meth-6-TridecylPiperidine**	281.528 g/mol	281.30825026 Da	C_19_H_39_N	C (81.06%), H (13.96%), N (4.98%)	Carvalho et al., 2019 [58]
**cis-2-Meth-6-PentadecylPiperidine**	309.582 g/mol	309.339550389 Da	C_21_H_43_N	C (81.47%), H (14.00%), N (4.52%)	Carvalho et al., 2019 [58]
**2-Dodecylsulfanyl-1,4,5,6-tetrahydropyrimidine**	284.51 g/mol	284.228620212 Da	C_16_H_32_N_2_S	C (67.55%), H (11.34%), N (9.85%), S (11.27%)	Uko et al., 2019 [62]
**[(dodecylsulfanyl)(methylamino)methyl](methyl)amine**	274.51 g/mol	274.244270277 Da	C_15_H_34_N_2_S	C (65.63%), H (12.48%), N (10.21%), S (11.68%)	Uko et al., 2019 [62]
**2-(dec-9-en-1-yl)-3-ethyl-1,3-oxazolidine**	239.403 g/mol	239.224914558 Da	C_15_H_29_NO	C (75.26%), H (12.21%), N (5.85%), O (6.68%)	Uko et al., 2019 [62]
**2-methyl-6-pentadecyl-2,3,4,5-tetrahydropyridine**	307.566 g/mol	307.323900324 Da	C_21_H_41_N	C (82.01%), H (13.44%), N (4.55%)	Yan et al., 2017 [59]
**2-methyl-6-tetradecyl-2,3,4,5-tetrahydropyridine**	293.539 g/mol	293.30825026 Da	C_20_H_39_N	C (81.84%), H (13.39%), N (4.77%)	Yan et al., 2017 [59]
**6-hexadecyl-2-methyl-2,3,4,5-tetrahydropyridine**	321.593 g/mol	321.339550389 Da	C_22_H_43_N	C (82.17%), H (13.48%), N (4.36%)	Yan et al., 2017 [59]
**2,4-dimethyl-6-nonadecylpiperidine**	379.717 g/mol	379.417800711 Da	C_26_H_53_N	C (82.24%), H (14.07%), N (3.69%)	Karlsoon et al., 2015 [63]

## 4. Discussion

### Summary of Evidence

It is clear from an initial examination of the literature that *Solenopsis invicta* is one of the most invasive insects, and where it has settled, it has caused difficulties for ecosystems [69] and the agricultural economy. Furthermore, it poses a risk to human health because anaphylactic shock is induced in sensitive individuals following stings, causing deaths every year from anaphylactic shock in the countries where it has established itself [44]. The eradication of *Solenopsis invicta* and *Solenopsis richteri* or their hybrids should be carried out where possible and must be strictly contained in its expansion and spread [70].

The increase in danger is also influenced by the location of the nests, which can be found in both agricultural and urban areas, as well as in residential areas. Consequently, attacks on children and infants are common [71], sometimes resulting in fatal outcomes [72].

In areas where *Solenopsis invicta* has established a stable settlement, the percentage of the population annually subjected to sting injury by this species varies from 30% (China) [73] to 40% (southeastern United States) [74]. Approximately 10% of these punctures lead to the development of fever and other symptoms, such as dizziness, hives, or other systemic reactions, including anaphylactic shock, with at least 32 deaths attributed to *Solenopsis invicta* stings in recent years in the USA [75].

This aggressive behaviour of RIFAs is also observed during floods and inundations. *Solenopsis invicta* is known to have the ability to create floating rafts for survival, with the entire colony involved in construction. A study conducted in 2023 reported how these rafts caused sting injuries to individuals on boats that accidentally came into contact with *Solenopsis invicta* rafts [76].

Therefore, *Solenopsis invicta* rafts that form during floods can further endanger individuals and rescuers working to save human lives, leading to an underestimation of additional health risks [76].

In some studies, the impact on the mental health of individuals subjected to *Solenopsis invicta* stings has been evaluated, with potential negative effects including the development of posttraumatic stress disorder (PTSD). The preliminary results of the study conducted by Wang et al. (2018) are inconclusive, with only 2 out of 46 subjects developing PTSD within 30 days. This aspect must be carefully considered in areas where *Solenopsis invicta* (RIFA) infestation affects households [77].

It is interesting to note how the presence of *Solenopsis invicta* can influence the epidemiology of seemingly unrelated pathologies. It has been hypothesized that the incidence of “α-Gal syndrome” (allergy to mammal meat), related to Lone Star tick (*Amblyomma americanum*) bites, may decrease in areas with ecological competition with *Solenopsis invicta*, as reported in a recent study by Wilson et al. (2021) [78].

The venom, which contains alkaloids, could have medical applications, as suggested for other venomous animal species. Specifically, the literature suggests a potential use for neoplastic diseases or as an antibacterial agent. As a result of this scoping literature review, 11 studies involving *Solenopsis invicta* venom or its derivatives were identified through databases, including 7 microbiological studies, 4 conducted on cell lines, and a single study on an animal model replicating the characteristics of psoriasis [64].

Initial studies on the composition of *Solenopsis invicta* venom were conducted by MacConnell et al. in 1970 [79] and in 1971 [80] (referred to as *Solenopsis saeuissima* in these studies, with the name *Solenopsis invicta* being accepted after 1972). These authors were the first to identify solenopsin A (trans-2-methyl-6-n-undecylpiperidine), the name of which was proposed, and subsequently identified all five major alkaloids that constitute 99% of the venom: trans-2-methyl-6-n-tridecylpiperidine (solenopsin B), trans-2-methyl-6-(cis-4-tridecenyl)-piperidine (dehydrosolenopsin B), trans-2-methyl-6-n-pentadecylpiperidine (solenopsin C), and trans-2-methyl-6-(cis-6-pentadecyl)-piperidine (dehydrosolenopsin C). Notably, Chen et al. (2009) [81] recently identified an additional piperidine alkaloid [81].

The composition of the venom of *Solenopsis invicta* is characterized by the predominance of piperidine alkaloids, in contrast to the high concentrations of peptides and proteins present in the venoms of bees and wasps.

It is hypothesized that one reason why the venom of *Solenopsis invicta* contains fewer proteins compared to the venom of other ants and more generally to that of other hymenopterans is that this species evolved more recently than more ancestral ant species, whose venom exhibits a higher protein content [82]. Most naturally occurring alkaloids have been widely described as originating from plant extracts [83]. This makes ants of the *Solenopsis* genus unique among other hymenopterans in their ability to produce large amounts of bioactive alkaloids. Furthermore, it is believed that these alkaloids are synthesized within the convoluted gland of the venom apparatus [84], rather than derived from the digestion of plant components [83].

The primary solenopsins, classified as A, B, C, and D, are distinguished based on the length of the alkyl side chain at position six of the piperidine ring (A: C11, B: C13, C: C15, and D: C17). However, various aspects of the biology of ants in the genus *Solenopsis* contribute to significant variability in the alkaloid and protein profile of their venom [85]. Each Solenopsis species exhibits a unique profile of piperidine alkaloids and proteins, with characteristic differences between *Solenopsis invicta*, *Solenopsis richteri*, and their hybrids [86]. The venom profile of these hybrids reflects a chemical combination of the two species.

Additionally, the venom composition varies according to the age and social role of individual ants, with differences observed among minor and major workers, alates, and queens, and depending on whether the colony is monogynous or polygynous in origin [87].

The discovery of new alkaloid and protein components in the venom of *Solenopsis invicta* is ongoing, and further studies are necessary to fully understand the venom’s composition and functions. Furthermore, the biosynthesis of alkaloids by *Solenopsis invicta* remains a relatively unexplored area, with limited research on the processes responsible for their production [85].

One of the earliest studies on the biological properties of *Solenopsis invicta* venom was conducted in the USA by Blum et al. [54] and was published in 1958. This study aimed to explore the potential of *Solenopsis invicta* venom as an insecticide and antibacterial agent, demonstrating interesting activity against the following bacteria: *Micrococcus pyogenes*, *Streptococcus pyogenes*, *Escherichia coli*, and *Lactobacillus casei* [54].

The authors explain how the lesions prove to be antiseptic upon inspection, if this statement could be true in an initial phase and in any case absolutely to be considered as one of the complications of pustules deriving from bites is secondary bacterial superinfection, which must be previously avoided with skin disinfection object of the injury [88].

In a study conducted in 2009 by Sullivan et al. [56], the growth of four bacteria was inhibited (*Streptococcus pneumoniae*, *Staphylococcus aureus*, *Enterococcus faecalis*, and *Stenotrophomonas maltophilia),* revealing the bacteriostatic action of the alkaloids present in the venom. In particular, the first three bacteria have clinical relevance and directly impact human health. For instance, *Enterococcus faecalis* is known for causing refractory endodontic lesions in the field of dentistry [89].

Additionally, Sullivan et al. reported [56], in contrast to Jouvenaz et al. (1972) [55], no bacteriostatic action against *Escherichia coli*. The venom of *Solenopsis invicta,* according to these early studies, appears to be more effective against Gram-positive bacteria than against Gram-negative bacteria, against which it showed only weak toxicity [55].

In particular, a study on Gram-negative bacteria was conducted by Park et al. in 2008 [57], who evaluated the cytotoxicity of solenopsin-A against *Pseudomonas aeruginosa*, an opportunistic pathogen implicated in different human diseases (endocarditis, urinary tract infections, skin infections, and medical complications in cystic fibrosis). Park reported that the alkaloids present in the venom did not inhibit growth in the first 8 h, suggesting that the inhibition of quorum-sensing (QS) signalling and the subsequent reduction in virulence factor production are due to the competition of solenopsin-A with C4-HSL, leading to the modulation of virulence factors and biofilm formation [57].

The inhibition of biofilm formation and adhesion was also investigated by Carvalho et al. (2019) for steel and polyester surfaces treated with solenopsins of *Solenopisis invicata* for healthcare and food use [58].

The results of the study revealed inhibition rates of 62.7% for *Pseudomonas fluorescens* on polyesters and 59.0% for *Pseudomonas fluorescens* on stainless steel surfaces. Furthermore, solenopsins drastically reduced the cell populations of mature biofilms that were already growing on untreated surfaces of polyester but not on stainless steel surfaces [58].

Biofilm formation is one of the main causes of urinary tract inflammation, rejection of surgically implanted prostheses, and dental plaque formation [90]. Therefore, the possibility of blocking its formation and growth represents an important target in the healthcare field [91,92].

Fungal effects have also been reported by Storey et al. on isolates of the genera *Beauveria bassiana*, AF-4 and 447, *Metarhizium anisopliae*, and *Paecilomyces fumosoroseus* [93]. In another recent study, solenopsis alkaloids were tested on fungi and bacteria (*Botrytis cinerea*, *Fusarium oxysporum*, *Phytophthora nicotianae*, *Phytophthora cryptogea*, *Pseudomonas syringae*, *Phytopythium citrinum, Rhizoctonia solani*, *Sclerotonia rolfsi, Xanthomonas axonopodis*, and *Xanthomonas campestris*). Naturally, these studies are of agronomic and environmental interest [94].

Interestingly, the antibacterial, antifungal, and antiprotozoal activities reported by Yan et al. (2017) [59] were achieved through the synthesis of piperidine alkaloids, starting from the antimicrobial activity of solenopsins. The pathogens under analysis were *Cryptococcus neoformans*, *Candida albicans*, and *Aspergillus fumigatus*, as well as antibacterial agents against methicillin-resistant *Staphylococcus aureus* (MRSA), vancomycin-resistant *Enterococcus faecium* (VRE), *Escherichia coli*, and *Pseudomonas aeruginosa*. The synthesized compounds were ineffective against *Aspergillus fumigatus*, MRSA, *Escherichia coli*, and *Pseudomonas aeruginosa* and showed antifungal activity against *Candida neoformans* and *Candida albicans* [59].

Both *Cryptococcus neoformans* and *Candida albicans* are opportunistic pathogens that cause diseases in humans, such as cryptococcosis and candidiasis, especially in immunocompromised individuals whose antifungal therapies do not always respond to pharmacological treatments [95].

Additionally, Yan et al. (2020) reported an inhibitory effect on *Leishmania donovani promastigotes* and *Trypanosoma brucei*, which are responsible for visceral leishmaniasis and human African trypanosomiasis, respectively [59]. It also seems to have an effect on *Trypanosoma cruzi*, which is responsible for Chagas disease, as reported by Silva et al. (2020) [60]. These recent studies provide interesting data on the potential use of solenopsins as new natural drugs against parasitic diseases caused by kinetoplastids [60].

Other applications of solenopsins, particularly solenopsin-A, are in the field of tumour pathology. In fact, Arbiser et al. (2007) [61] discovered that it is a potent angiogenesis inhibitor and reported promising data from in vivo models of embryonic zebrafish and renal carcinoma tumour cell lines [61].

Recent studies on cell lines have suggested that the trans isomers of solenopsin-A, which are more potent than the corresponding cis isomers, inhibit the phosphatidylinositol-3-kinase (PI3K) signalling pathway in upstream PI3K cells. PI3K and its effectors (Akt) play crucial regulatory roles in controlling apoptosis, proliferation, and angiogenesis [96]. These assumptions are partially confirmed by a study conducted by Uko et al. (2019) [62] on a solenopsin analogue executed on human lung tumour cells and WB-ras rat liver epithelial cells. The analogue of solenopsin reduced the phosphorylation of Akt at the activation site Thr308 and the main downstream effectors of Akt kinase without directly inhibiting Akt kinase. PI3K and Akt are amplified or overexpressed in various neoplasms, and their inhibition induced by solenopsins could hinder tumour growth [97,98].

The trans isoform of solenopsin-A exhibits biological activity similar to that of ceramide in human melanoma cells. Therefore, it may be beneficial in the treatment of hyperproliferative conditions and could be useful in the treatment of neoplastic and hyperproliferative skin conditions [63].

In fact, solenopsin was also evaluated by Arbiser et al. in 2017 in a murine model of psoriasis. Compared with control mice, mice treated with solenopsin analogues for 28 days showed significant decreases in acanthosis and hyperkeratosis, decreases in TLR4 expression and IL-22, and increases in IL-12 [64]. This model of acanthosis and hyperkeratosis is very similar to the pathological changes observed in human psoriasis. By restoring ceramide signalling, solenopsin can potentially restore normal skin homeostasis through the anti-inflammatory AP-1/IL-12 pathway [64].

**Figure 6:**
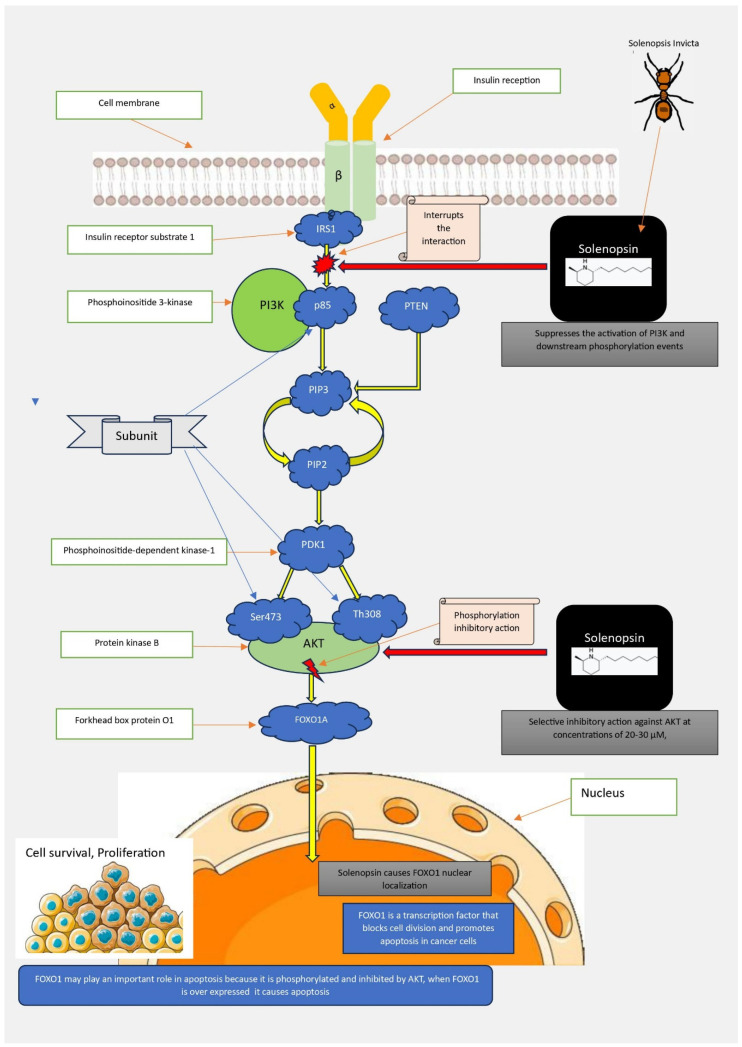
Mechanistically, solenopsin has an in vitro selective inhibitory effect on AKT at concentrations of 20–30 μM; furthermore, it is hypothesized that solenopsin interrupts the interaction between IRS1 and the p85 regulatory subunit of PI3K and that it inhibits the activation of PDK1. IRS1 (insulin receptor substrate 1), PI3K (phosphoinositide 3-kinase), PDK1 (phosphoinositide-dependent kinase-1), AKT (protein kinase B), FOXO1A (Forkhead box protein O1).

The antitumour and antiproliferative effects are thought to arise from the inhibitory action of solenopsin, which suppresses the activation of PI3K and downstream phosphorylation events, such as the phosphorylation of Akt and FOXO1A (an Akt substrate). Specifically, the inhibitory action occurs downstream of IRS1, presumably by interrupting the interaction between IRS1 and the regulatory p85 subunit of PI3K. Moreover, the selective in vitro inhibition of Akt by solenopsin may represent a significant pharmacological target given the limited development of Akt inhibitors (Figure 6) [62].

The alkaloids present in the venom of *Solenopsis invicta*, including solenopsin and its derivatives, can be readily synthesized on a large scale. The presence of a free secondary amino group allows them to be conjugated with other molecules, enabling targeted release. This conjugation could therefore offer a novel therapy for advanced-stage neoplasms or proliferative disorders, such as psoriasis [64].

However, more comprehensive studies and a thorough evaluation of safety and efficacy are needed before considering practical applications in healthcare.

The findings of Alejandro Peralta Soler, published in an article in “INTERALIA MAGAZINE”, are also interesting and fascinating, in which he described behavioural analogies between the migration and survival mechanisms of *Solenopsis invicta* and the metastatic potential of tumours [99]. The aggregation of tumour cells within fluids provides a safer format for the protection of cells with reproductive potential and, ultimately, for survival and metastatic tumour growth. This could serve as a translational inspiration and suggestion for the study of tumour cell behaviour in the liquid environment of lymphatic and blood vessels [99].

A recent study by dos Santos Pinto et al. [100] identified 46 distinct proteins and peptides in *Solenopsis invicta* venom, categorized into four functional groups: true venom components, housekeeping proteins (proteins from the venom gland without apparent toxic roles), muscle proteins (proteins originating from the ant’s body muscles, such as Prominin-like protein and Troponin C, likely released inadvertently during venom collection), and proteins involved in chemical communication [100].

**Figure 7:**
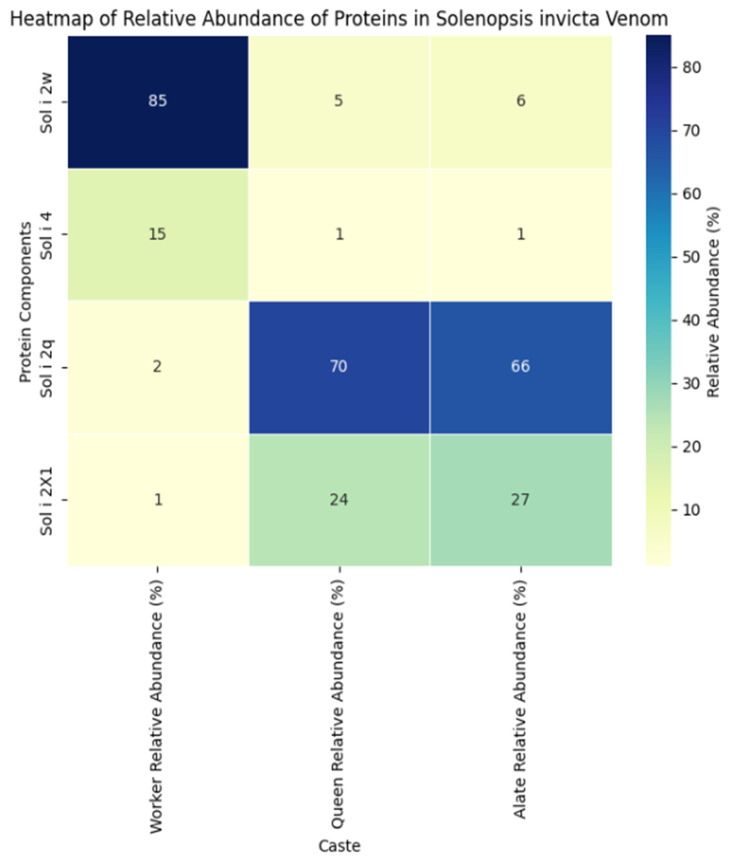
Heatmap of relative abundance %. Data were extracted and estimated from Figure 2 of the study by Das et al. (2018) [101] using Python 3.13.0 and JupyterLab. These values pertain to the proteins “Sol i 2w,” “Sol i 4,” “Sol i 2q,” and “Sol i 2X1” found in the poison sacs of workers, queens, and alates.

The group designated as “true venom components” includes 21 distinct proteins, among which Sol 1–4 are recognized as potent allergens, specifically including Sol i 2w, Sol i 4, Sol i 2q, and Sol i 2X1 (see Figure 7) [101]. The most abundant protein in this group is a pseudochetoxin, similar to those found in centipedes. Among these peptides and proteins, we find the following: phospholipases A1 and A2, which disrupt cell membranes, facilitating venom spread and causing tissue damage; snake-like myotoxins, which induce muscle necrosis and increase vascular permeability; and disintegrins and metalloproteinases, which provoke hemorrhage and necrosis. Neurotoxins, such as U5-ctenotoxin Pk1a and Tc48a, block sodium channels, leading to paralysis. Additionally, antimicrobial peptides, such as ponericins, protect the colony from infections. Finally, the atrial natriuretic peptide (ANP) lowers blood pressure in victims, facilitating venom spread. Together, these lethal and harmful components function both in colony defence and in prey immobilization, while also maintaining asepsis [102].

**Figure 8:**
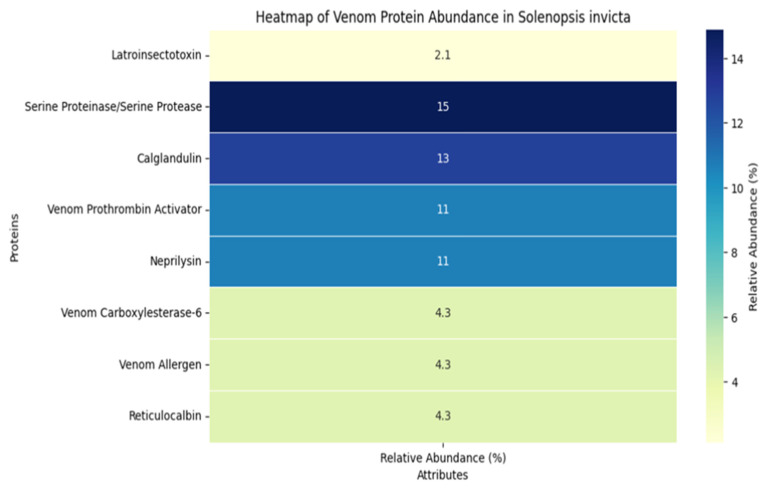
Heatmap of relative abundance %. Data were extracted and estimated from Figure 3b and information reported in the study by Cai et al. (2022) [103]. The heatmap was generated using the extracted data with Python 3.13.0 and JupyterLab, and it pertains to the proteins and peptides in the S*olenopsis* invicta sample.

Through a comprehensive transcriptomic and proteomic analysis of *Solenopsis invicta* by Cai et al. in 2022 [103], 47 proteins were identified (1 more than reported by dos Santos Pinto et al. [101]). Among the most prominent proteins emerging from the transcriptome and subsequently detected in the proteome are Latroinsectotoxin (2.13%), Serine proteinase/serine protease (14.89%), Calglandulin (12.77%), Venom prothrombin activator (10.64%), Neprilysin (10.64%), Venom carboxylesterase-6 (4.26%), Venom allergen (4.26%), and Reticulocalbin (4.26%) (see Figure 8). The primary techniques used to identify the largest number of peptides in Solenopsis venom, as detailed by dos Santos Pinto et al. [2], included Two-Dimensional Gel Electrophoresis (2-DE), MALDI-TOF/TOF Mass Spectrometry, and LC-IT/TOF-MS and MSn (Ion Trap and Time-of-Flight Mass Spectrometry) [101,103].

In terms of potential medical and pharmacological applications, certain peptides, such as phospholipases and allergens, exhibit immunological effects that could be explored for allergen desensitization therapies. Antimicrobial proteins, such as ponericins, with activity against both Gram-positive and Gram-negative bacteria, present promising potential for antimicrobial therapies. Additionally, neurotoxic components, such as U5-ctenotoxin and other paralytic toxins [104], could be investigated for the development of new neuroactive compounds.

Peptides with cytotoxic or neurotoxic properties (such as Latroinsectotoxin [105]) may also offer therapeutic avenues for pain relief or other neurological conditions, leveraging their specific activity against ion channels or receptors. However, clinical applications are constrained by the challenges of venom extraction and purification, which complicate research and pharmacological development [106].

Based on current knowledge, there is no direct evidence of therapeutic efficacy in human subjects. The only studies conducted on humans are epidemiological studies on the prevalence of red imported fire ant stings in infested areas. In this regard, a recent systematic literature review conducted by Lopez et al. [107] reported a prevalence as high as 51% in Texas (USA) in 1995 [108], while the most recent study conducted by Liu et al. in 2021 [109] in Taiwan involving 10,127 participants reported a 12-month prevalence of stings of 37.7% [109].

Bringing *Solenopsis invicta* venom-derived compounds into clinical settings remains a distant prospect for several reasons. Currently, research on the venom’s therapeutic effects is mainly limited to in vitro studies [110] or animal models, and advancing to clinical trials will require further evidence of safety and efficacy in humans. Key challenges include the limited data on the selective toxicity of these compounds towards healthy human cells, a critical aspect for therapeutic development. Some progress has been made, such as the synthesis of solenopsin analogues to study toxicity and improve selectivity; however, these compounds require further investigation to confirm their stability and bioavailability in clinical environments. A detailed understanding of the venom’s selectivity for targeting cancer cells over healthy cells is essential. At present, it remains unclear how safe these compounds are for therapeutic use in humans, as no direct clinical studies have been conducted. The next steps include developing more refined preclinical models and employing in silico tools to predict toxicity and interactions with human cells [111]. However, a comprehensive assessment of safety and efficacy remains fundamental before advancing to clinical trials [111].

The lack of studies conducted on humans in the literature thus represents a limitation of this literature review, as there are no real proofs of potential uses in the medical or oncological fields beyond its use as an antibacterial agent. Only the presence of indications suggests potential future applications, which need to be validated by further in vitro studies and subsequently in clinical trials.

## 5. Conclusions

Our findings demonstrate that *Solenopsis invicta* venom possesses significant therapeutic potential, particularly due to its unique blend of bioactive alkaloids and peptides. Components such as solenopsin and various allergenic peptides (Sol i 1–4) have shown properties relevant to antimicrobial, antifungal, and even antitumour applications, marking them as promising candidates for drug development. The venom’s neurotoxic peptides, like U5-ctenotoxin, have potential applications in managing chronic pain and neurological disorders due to their interaction with ion channels and receptor sites, an approach that aligns with current pharmacological interests in ion channel modulators.

Despite the considerable promise of these bioactive components, our analysis highlights significant challenges. The complexity of venom extraction and the purification processes required to isolate these proteins for clinical use impose substantial obstacles. Additionally, toxicity concerns, particularly with neurotoxic and cytotoxic components, necessitate thorough evaluation of safety and efficacy through preclinical models.

In line with these observations, future studies should focus on detailed mechanism-of-action analyses of individual venom components, particularly their interactions with bacterial membranes, tumour cells, and neural ion channels. Additionally, advanced in silico modelling for toxicity prediction could provide insights for safer pharmacological applications, reducing the risk associated with clinical translation. The development of analogues or synthetic versions of venom peptides could also help overcome limitations related to toxicity and bioavailability.

Thus, while *Solenopsis invicta* venom holds substantial promise as a source of novel bioactive compounds, significant research efforts are essential to translate these findings into viable therapeutic applications. This study not only expands our understanding of Solenopsis venom’s composition but also highlights its potential impact on medical and pharmaceutical research, particularly in developing next-generation antimicrobials, analgesics, and anticancer agents.

## Data Availability

Not applicable.

## Supplementary Materials

The following supporting information can be downloaded at: https://www.mdpi.com/article/10.3390/biom14121499/s1, Supplementary Materials S1: Additional International Union of Pure and Applied Chemistry (IUPAC) chemical formulas of the alkaloids present in the venom of *Solenopsis invicta*; Supplementary Materials S2: INPLASY (International Platform of Registered Systematic Review and Meta-analysis Protocols) workflow [53,112].
