# Supplementary material for: Therapeutic Potential of Solenopsis invicta Venom: A Scoping Review of Its Bioactive Molecules, Biological Aspects, and Health Applications
Source: Biomolecules. 2024 Nov 24;14(12):1499. doi: 10.3390/biom14121499 (PMC11673062; doi:10.3390/biom14121499)
Supplement: Supplementary file 1 [file biomolecules-14-01499-s001.zip › biomolecules-3244058-supplementary.pdf]

cis-2-Meth-6-undecyl piperidine	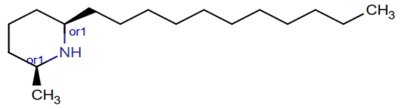
trans-2-Meth-6-undecyl piperidine	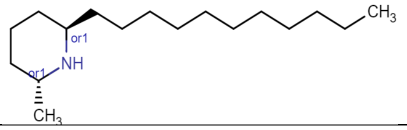
trans-2-Meth-6-tridecenylpiperidine	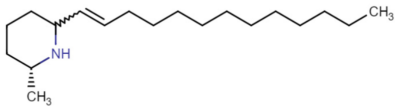
trans-2-Meth-6-tridecyl piperidine	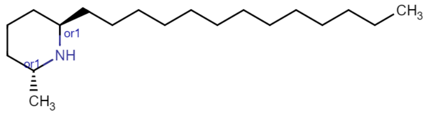
trans-2-Meth-6-pentadecenylpiperidine	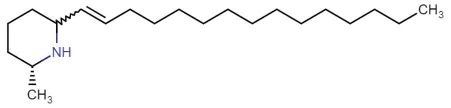
trans-2-Meth-6-pentadecylpiperidine	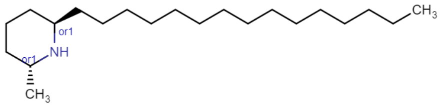
cis-2-Meth-6-TridecylPiperidine	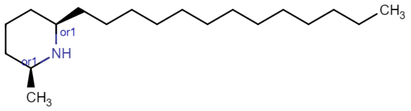
cis-2-Meth-6-PentadecylPiperidine	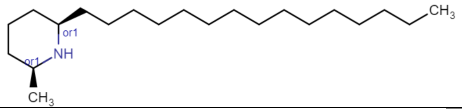
2-Dodecylsulfanyl-1,-4,-5,-6-tetrahydropyrimidine	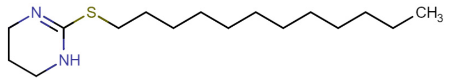
[(dodecylsulfanyl)(methylamino)methyl](methyl)amine	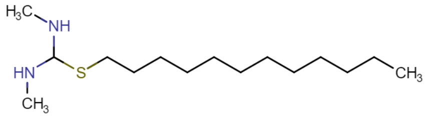
2-(dec-9-en-1-yl)-3-ethyl-1,3-oxazolidine	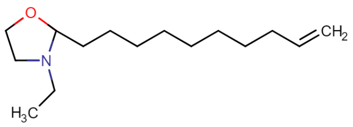
2-methyl-6-pentadecyl-2,3,4,5-tetrahydropyridine	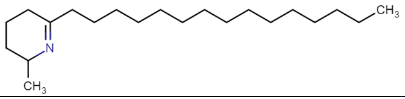
2-methyl-6-tetradecyl-2,3,4,5-tetrahydropyridine	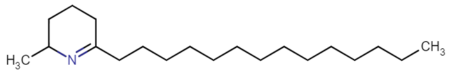
2-methyl-6-hexadecyl-2,3,4,5-tetrahydropyridine	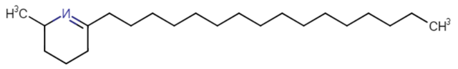
2,4-dimethyl-6-nonadecylpiperidine	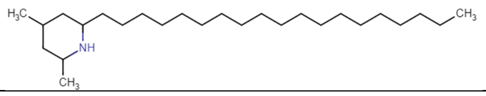

Supplementary Materials S1

The figures of the molecules were obtained via the use of the online software https://chemicalize.com/app/calculation on 16 June 2024 starting from the IUPAC formula.

Supplementary Materials S2

The scoping review was conducted following the PRISMA-ScR checklist (PRISMA Extension for Scoping Reviews), as outlined by Tricco et al. [1]. Although the review protocol was prepared before the database search was executed, it was deemed appropriate not to register it. The reason for choosing not to register the review lies in the nature of scoping reviews, which are literature reviews that pose broader research questions than systematic reviews and aim to examine the breadth and scope of a topic rather than answering specific research questions or obtaining predetermined outcomes. For these reasons, scoping reviews and mapping reviews cannot be registered on the PROSPERO platform [2]. The scoping review was alternatively registered on INPLASY (International Platform of Registered Systematic Review and Meta-analysis Protocols) under the registration number INPLASY202490103, with the DOI: 10.37766/inplasy2024.9.0103

1. Tricco, A.C.; Lillie, E.; Zarin, W.; O'Brien, K.K.; Colquhoun, H.; Levac, D.; Moher, D.; Peters, M.D.; Horsley, T.; Weeks, L. PRISMA extension for scoping reviews (PRISMA-ScR): checklist and explanation. *Annals of internal medicine* **2018**, *169*, 467-473.
2. Schiavo, J.H. PROSPERO: an international register of systematic review protocols. *Medical reference services quarterly* **2019**, *38*, 171-180.
